# The Snake Dance

**Published:** 1885-10

**Authors:** 


### The Snake Dance.

A CARNIVAL OF HORRORS AMONG THE MOQUL INDIANS OF ARIZONA.

On the morning of August 18th the great dance commenced. Quite a number of spectators, including two ladies, had gone up from Navajo springs, but during all the proceedings the presence of this company was utterly ignored, so completely were the savages absorbed in their terrible carnival. In one corner of the open court where the ceremony took place was a conical stone rising from the ground, and around this about 30 of the Indians marched, chanting in a weird, lugubrious fashion. After this the entire body of about 130 marched over a number of loose planks upon which "sacred meal" had been strewn by a number of squaws especially set apart to do this work and who wore white mantles and had their long black hair done up in enormous wagon wheel rolls. The bucks wore a tunic coming down about midway to the thighs, moccasins upon the feet, and attached to the naked calf of the right leg of each one was the shell of a terrapin, within which were pebbles or seed of some kind that kept up a constant rattling. A handkerchief was bound about the brow of each one, and their faces were painted in a way to create a startling effect. The upper half of the faces was painted a deep black and the lower half white. The grotesque mass of creatures, after passing over the sacred meal, then arranged themselves in a column of twos to commence the snake dance proper. An attendant, armed with two eagle feathers, was detailed to furnish snakes to every two men, and with the procession thus completed, it commenced to circle around the snake den. As each pair would pass the attendant would thrust his naked arm in the den, seize a writhing serpent and jerk it out. It was then handed to the buck who would grasp the body of the reptile about midway between his teeth. In this way every man in the procession was furnished with a snake, and when the horrid equipment was complete they would gyrate around in a circle, flinging their arms and legs high in the air, and wagging their hideous heads, but unable to make any noise, because their mouths were full of snakes. It was an awful spectacle, those painted wretches with their mouths full of writhing rattlesnakes, and the ground at their feet alive with the reptiles, that all the while kept up a furious rattling, but for some inexplicable reason refused to bite the men who were ruffling their tempers to such a degree. During the dance one of the reptiles sank his fangs into the cheek of an Indian who had just picked him up, but another Indian stepped forward, pulled the reptile loose, and the one who had been bitten went on with the dance, with his equanimity quite undisturbed. This part of the programme was protracted through half an hour, and then the snakes were all thrown together in a writhing mass upon a piece of ground that had been sprinkled with sacred meal, where they were kept together until the bucks could divide themselves into four squads of equal number. At a given signal each squad rushed upon the mass of serpents, and each man seizing all of them he could possibly hold in each hand, bounded off like a deer to the crest of the butte, made his way down the face of the cliff with marvellous rapidity, and at the imminent risk of his life, for a misstep would hurl him 500 feet, and when all of the 130 painted bucks, with their hands full of squirming serpents, had reached the bottom, they started off without halting an instant, racing across the plains almost with the fleetness of a deer, separating into four groups, going north, south, east and west. When they were out a distance of about half a mile the snakes were turned loose among the rocks, and the Indians, relieved of their pets, wheeled around and without stopping raced back to the butte, climbed panting up the steep sides and disappeared one at a time into the cavernous depths of the estufa a great chamber hollowed out of solid rock.

